# Subcellular-Level Mitochondrial Energy Metabolism Response in the Fat Body of the German Cockroach Fed Abamectin

**DOI:** 10.3390/insects13121091

**Published:** 2022-11-27

**Authors:** Lin-Yu Yang, Xiao-Jie Yang, Zi-Shun Zhao, Qi-Lin Zhang

**Affiliations:** Faculty of Life Science and Technology, Kunming University of Science and Technology, Kunming 650500, China

**Keywords:** fat body mitochondria, *Blattella germanica*, abamectin feeding, energy metabolism, toxic response

## Abstract

**Simple Summary:**

To date, it is rare to study the effects of environmental toxicants (e.g., abamectin, a widely used pesticide) on mitochondrial energy metabolism in vitro from a subcellular level in insects, especially from an omics perspective (e.g., metabolomics) due to insufficient samples of mitochondria. The fat body of the German cockroach (*Blattella germanica*) plays an important role for detoxification, with responses of critical subcellular components (e.g., mitochondria) to abamectin. When *B. germanica* were fed abamectin, their in vitro mitochondrial morphology was affected, with changes in activity of key enzymes involved in energy metabolism. Furthermore, metabonomic analysis of in vitro mitochondria of *B. germanica* uncovered a set of metabolites and their pathways related to mitochondrial energy metabolism in response to abamectin feeding/stress. These molecules and pathways were primarily involved in ATP production and energy consumption including oxidative phosphorylation, TCA cycle, and pentose phosphate pathway. The current work is a good example of investigations on subcellular toxicology from multiple perspectives (morphology, physiology, and metabolomics).

**Abstract:**

Mitochondria are the leading organelle for energy metabolism. The toxic effects of environmental toxicants on mitochondrial morphology, energy metabolism, and their determination of cell fate have already been broadly studied. However, minimal research exists on effects of environmental toxicants such as pesticides on mitochondrial energy metabolism at in vitro subcellular level, particularly from an omics perspectives (e.g., metabolomics). Here, German cockroach (*Blattella germanica*) was fed diets with (0.01 and 0.001 mg/mL) and without abamectin, and highly purified fat body mitochondria were isolated. Swelling measurement confirmed abnormal mitochondrial swelling caused by abamectin stress. The activity of two key mitochondrial energy metabolism-related enzymes, namely succinic dehydrogenase and isocitrate dehydrogenase, was significantly affected. The metabolomic responses of the isolated mitochondria to abamectin were analyzed via untargeted liquid chromatography/mass spectrometry metabolomics technology. Fifty-two differential metabolites (DMs) were identified in the mitochondria between the 0.001 mg/mL abamectin-fed and the control groups. Many of these DMs were significantly enriched in pathways involved in ATP production and energy consumption (e.g., oxidative phosphorylation, TCA cycle, and pentose phosphate pathway). Nineteen of the DMs were typically related to energy metabolism. This study is valuable for further understanding mitochondrial toxicology under environmental toxicants, particularly its subcellular level.

## 1. Introduction

The German cockroach (Blattodea: Blattellidae: *Blattella germanica*) is a common indoor pest. This lineage transmits various pathogenic microorganisms and parasites that cause diseases including cholera, diarrhea, and dysentery [1,2]. Because of its rapid development, high fecundity, and strong adaptability, *B. germanica* has been an important pest control target indoors [2]. At present, chemical insecticides such as abamectin are the most dominant ones used for controlling *B. germanica* [3]. However, its abuse, along with environmental toxicity, causes *B. germanica* to develop insecticide resistance and even exerts a passive influence on human health [2,4]. In order to manage resistance and successfully control this pest, a thorough study on the effects of insecticides (e.g., abamectin) on *B. germanica* is essential.

Abamectin (Abamectin B1, MK-936), which is a macrocyclic lactone compound, has proven to have extraordinarily potent insecticidal and antihelmintic activities [5,6]. Concerns regarding the safety of abamectin have risen with its broad use across the world. The United States Environmental Protection Agency has announced that abamectin is highly toxic to fish and bees, and extremely toxic to mammals and aquatic invertebrates [5,7]. To date, abamectin has been tested against a variety of urban pests such as *B. germanica*. In particular, insecticide resistance, effective concentration of abamectin and the behavior response of *B. germanica* have been primarily investigated [3,8,9]. However, little is known about the mechanisms of the toxic response to abamectin stress in *B. germanica* and even animals.

Insect fat body has been widely considered to have a similar function to the liver of vertebrates [10], which participates in energy storage and homeostasis [11]. In particular, the fat body contains various enzymes needed for detoxification, and thus it is an important place for insect detoxification (detoxification activity of the fat body is second only to midgut). In addition, trophocytes, as primary functional cells in metabolism and storage in the fat body, contain abundant mitochondria [12]. The most direct mechanism of fat body toxicity at the subcellular level is the specific interaction of the toxicant with a critical subcellular component (e.g., mitochondria) [13,14]. Therefore, the fat body and therein mitochondria are important for detoxification response of *B. germanica*.

Mitochondria are one of the primary targets for toxic injury, leading to dysfunction due to energy imbalance, excessive reactive oxygen species, and/or apoptosis [15]. Previous studies showed that pesticides (e.g., baytex, k-othrine, and dimilin) caused inhibition of the succinate dehydrogenase (SDH) as key mitochondrial enzymes in the bug *Diplonychus indicus* [16]. Arsenicals decreased two metabolites (i.e., fructose-1,6-bis-phosphate and lactate) shared by glycolysis and gluconeogenesis as well as two metabolites (i.e., succinate and fumarate) in the tricarboxylic acid (TCA) cycle [17]. Ammonia nitrogen exposure caused mitochondrial structural damage in the gill of the clam *Ruditapes philippinarum* [18]. These observations suggest that mitochondria participate in the toxic response. In addition, the high-purity/isolated mitochondria from tissues are necessary for more accurately uncovering mitochondrial function [19,20,21], which is a basis for studying structural integrity, biological mechanisms, and functional competence of mitochondria at subcellular (organelle) level upon toxic stress [22]. Changes and responses in the isolated mitochondria can be more sensitively and accurately observed by comparing them with mitochondria enclosed by cytoplasm [21,23,24].

As the “energy factories” of animals, mitochondria generate 95% of their energy by oxidative phosphorylation (OXPHOS) and play important roles in energy metabolism [25,26]. In vertebrates, hepatic cells contain the largest number of mitochondria (1000–2000 mitochondria per cell) [27,28]. Several typical and key biological molecules have been related to energy metabolism in mitochondria such as enzymes (e.g., SDH and isocitrate dehydrogenase (IDH)) [29,30], metabolites (e.g., α-ketoglutaric acid (α-KG), adenosine 5′-monophosphate (AMP), and nicotinamide adenine dinucleotide (NADH)) [30,31], and metabolic pathways (e.g., TCA cycle, amino acid biosynthesis, and OXPHOS) [30,32]. These energy metabolism-related terms have particularly been linked to toxic responses to drugs at the organ, tissue, and cell level. For example, doxorubicin caused lesions in the electron transport system of the skeletal muscle, resulting in a loss of the contractile function of the tissues [33]. Aconitine induced abnormal cellular energy metabolism, which led to the decrease of ATP production and proton leak in human neuroblastoma cells [34]. In addition, despite that abamectin has been found to affect the bioenergetics of the isolated liver mitochondria in rat [5], the study was primarily performed at the physiological level, lacking other perspectives. Therefore, it remains unclear that environmental (non-drug) toxicants affect energy metabolism-related toxic responses of mitochondria at the subcellular level, particularly from various perspectives (e.g., morphology, physiology, metabolites, and metabolic pathways). This limitation obstructs a complete and correct knowledge of the role of mitochondria in the toxic response.

Herein, the mitochondria isolated from the fat body of abamectin-free (control) and abamectin-fed *B. germanica* (treatment) were obtained. We confirmed changes in the swelling produced by abamectin feeding, and the activities of SDH and IDH were measured to determine the effects of abamectin on key metabolism-related enzymes in mitochondria. Based on these effect confirmations, non-targeted/global metabolomics based on liquid chromatography/mass spectrometry (LC-MS) was employed to analyze differences in metabolite profiles of the control and treatment groups. Finally, we investigated effects of abamectin feeding on the enzymes, metabolites, and metabolic pathways associated with energy metabolism of the fat body mitochondria.

## 2. Materials and Methods

### 2.1. Sampling and Abamectin Feeding

Specimens of *B. germanica* were obtained came from the Health and Quarantine Office of Chongqing Customs, China. These specimens originate from colonies that have been kept indoors in isolation for about 25 years and have never been exposed to pesticides. Healthy individuals were selected for further experiments. Rearing methods were obtained from previous studies [35]. Briefly, insects were maintained in 3.8 L plastic containers held in a reach-in environmental chamber at 25 ± 1 °C under a 12-h light: 12-h dark photoperiod. A total, 600 adult males of 4 weeks were divided into three groups. The rearing unit contained corrugated cardboard harborages, a water source, and a standard bait base (Purina 5001 Rodent Diet, PMI Nutrition International, St. Louis, MO, USA).

The standard bait base was prepared as previously described [36]. A standard solution of abamectin in methanol (10 μg/mL, CAS 71751-41-2; Aladdin Bio-Chem Technology, Shanghai, China) was purchased. Two concentrations (i.e., 0.01 and 0.001 mg/mL) of abamectin solution were formulated on the standard bait base. The experimental concentrations were chosen according to prior studies [36,37]. Diets containing abamectin at these two concentrations can adequately induce gut microbial responses and *B. germanica* toxicity. The experimental individuals were fed approximately 0.5 g of abamectin-containing diets in Petri dishes. Feeding was conducted every 12 h for the two abamectin-fed groups. Every time before feeding, the previously remaining diets and feces were removed. As for the control groups, an equal amount of baits in methanol was employed to perform independent experiments in parallel. Each individual in the three groups (the control, 0.01 mg/mL, and 0.001 mg/mL abamectin-fed groups) was dissected using sterile scissors and tweezers at 1, 3, 7, 10, and 15 days post-feeding (dpf), and then white flocculent tissue (i.e., the fat body) that flows from the abdominal cavity was collected using a pipette, transferred to 2-mL EP tube, respectively. The collected samples were used to isolate mitochondria.

### 2.2. Mitochondria Isolation, Western Blot, and Sample Division

Mitochondria isolation and subsequent Western blot analysis used to confirm the purity of the isolated mitochondria were performed following methods recently developed by us [21]. The detailed procedure is included in the Supplementary Material. The mitochondria isolated from the fat body samples collected at 3, 7, and 15 dpf were used to examine the swelling and the ultrastructure, and the enzymatic activity of SDH and IDH was tested using mitochondria obtained at 1, 3, 7, 10, and 15 dpf. The experiments were independently conducted in triplicate (n = 3).

The mitochondria isolated from the fat body of *B. germanica* fed with 0.001 mg/mL abamectin and the control groups collected at 1, 3, 7, 10, and 15 dpf were equally mixed into a 2-mL EP tube. The experiments were independently conducted seven times (n = 7) for downstream metabolic analysis. Despite the mixture of collection at each dpf leading to a loss of information regarding the temporal level of metabolites at each day, the study aimed at an overview of what metabolites exhibit a response to abamectin stress. Therefore, it is acceptable for a loss of dynamic information of metabolite levels across different time points to occur in this study, as similarly adopted in recent publications [38,39].

### 2.3. Determination of Mitochondrial Swelling

To quantify the mitochondrial swelling degree, absorbance changes of mitochondria were monitored, as previously described [18,40]. Briefly, the isolated mitochondria (50 μg) were incubated at 37 °C for 10 min in a 200 μL STE solution on a 96-well plate. The absorbance was then monitored at 540 nm every 30 s until 10 min using a Varioskan LUX multimode microplate reader (Thermo Scientific, Waltham, CA, USA). The maximum value of ΔA540 per min and per mg protein was used to present the results of mitochondrial swelling. The experiments were repeated independently in triplicate.

### 2.4. Measurement of SDH and IDH Activities

SDH activity was determined using the SDH Test kit (Cat. No. 20210915, Solarbio, Beijing, China). Briefly, 0.1 g of the isolated mitochondria were mixed with 1 mL reagent I 10 μL reagent II, ground, and centrifuged at 11,000× *g* for 10 min at 4 °C. The supernatant was collected to measure SDH activity at 600 nm using a Varioskan LUX multimode microplate reader (Thermo Scientific). The SDH enzyme activity was assessed by consuming 1 nmol 2,6-DCPIP per mg of tissue protein per minute (U/mg prot). The experiments were repeated independently in triplicate.

The IDH activity was evaluated using the IDH Test kit (Cat. No. 20211013, Solarbio, China). Briefly, 0.1 g of the isolated mitochondria were mixed with 1.5 mL extract I 15 μL extract II, ground, and centrifuged at 1000× *g* for 10 min at 4 °C. The supernatant was collected, centrifuged at 11,000× *g* for 15 min at 4 °C, and added to 600 μL extract III and 15 μL extract II for ultrasonic crushing (power 40%, ultrasonic for 5 s, interval of 9 s, 4 min). The absorbance of the samples at 505 nm was measured after a 5 min reaction. The IDH enzyme activity was assessed by the production of 1 nmoL α-KG per mg tissue protein per minute (U/mg prot). The experiments were repeated independently in triplicate.

### 2.5. Metabolite Extraction

The isolated mitochondria were adequately lysed in 500 μL 50% methanol, vortexed for 1 min, incubated at room temperature for 10 min, stored overnight at −20 °C, and centrifuged at 13,000× *g* for 20 min at 4 °C. The supernatants were transferred to 96-well plates for further LC-MS analysis. To evaluate the system conditioning and quality control process, a pooled quality control (QC) sample was prepared by mixing equal volumes (10 μL) of each experimental sample.

### 2.6. LC-MS Analysis

The samples were analyzed by the LC-MS system following the instructions of the instrument. Chromatographic separations were performed using an ultra-performance liquid chromatography (UPLC) system (SCIEX, Warrington, UK). An ACQUITY UPLC T3 column (100 mm × 2.1 mm, 1.8 μm, Waters, Wilmslow, UK) was used for the reversed-phase separation. The mobile phase of solvent A consisted of water and 0.1% formic acid and that of solvent B consisted of acetonitrile, 0.1% formic acid. The gradient elution conditions were as follows: 0 to 0.5 min, 5% B; 0.5 to 7 min, 5% to 100% B; 7 to 8 min, 100% B; 8 to 8.1 min, 100% to 5% B; and 8.1 to 10 min, 5% B. The injection volume for each sample was 4 μL, and the flow rate was 0.4 mL/min. The column oven was maintained at 35 °C.

A high-resolution tandem mass spectrometer TripleTOF5600plus (SCIEX, UK) was used in both positive ion modes (PIM) and negative ion modes (NIM) to detect metabolites eluted from the column. The quadrupole time-of-flight (Q-TOF) was operated in both PIM and NIM. The curtain gas of the ion source was set at 30 PSI, the gas1 was set at 60 PSI, the gas2 was set at 60 PSI, and the interface heater temperature was 650 °C. The ion spray voltage floating was set at 5000 V and −4500 V for the PIM and NIM, respectively. The mass spectrometry data were acquired in information-dependent acquisition (IDA) mode. The detection was carried out over a mass range of 60 to 1200 *m*/*z*.

### 2.7. Metabolic Data Processing

The raw data files generated by LC-MS analysis were preprocessed by Progenesis QI (Waters Corporation, Milford, CT, USA), a specialized LC-MS data analysis software (https://www.nonlinear.com/progenesis/qi/, accessed on 12 December 2021), to conduct baseline filtering, peak identification, integration, retention time correction, and peak alignment. A three-dimensional matrix containing retention time (RT), mass-to-charge ratio (*m*/*z*) values, and peak intensity was obtained for each ion. Subsequently, the characteristic peak was searched in in-house and online using KEGG (Kyoto Encyclopedia of Genes and Genomes, http://www.genome.jp/kegg/, accessed on 25 December 2021) and HMDB (Hydrogen Mitigation Design Basis, http://www.hmdb.ca/, accessed on 25 December 2021) databases to annotate their function by matching their *m*/*z* values (MS, first mass spectrometry) and amino acid composition (MS/MS, secondary mass spectrometry). The reliable weight error of *m*/*z* values was less than 10 ppm; meanwhile, the metabolites were further determined according to matching scores of MS/MS. Only metabolites with a score of 700 or higher were considered valid. Furthermore, the remaining metabolites detected in less than 50% of QC samples or 80% of experimental samples were discarded to avoid false positive. Feature intensities were normalized using the probabilistic quotient normalization (PQN) algorithm in metaX software (version 1.4.16 ) to obtain the relative peak area [41]. The coefficient of variation (CV) of the relative peak area was then calculated for all QC samples. The metabolites with a CV greater than 30% were removed for further analysis. Relative metabolite quantification was performed by peak integration using mean normalized spectra after the removal of water, lipids, and contaminations.

The preprocessed data from PIM and NIM were imported into SIMCAP software (version 13.0) (Umetrics, Umeå, Sweden) to conduct unsupervised principal component analysis (PCA) and supervised partial least squares discriminant analysis (PLS-DA). Moreover, 200 permutation tests were performed to assess the quality of the PLS-DA model based on the following parameters: R^2^ (reliable goodness of fit, R^2^ > 0.5) and Q^2^ (reliable goodness of prediction, Q^2^ < 0).

### 2.8. Identification and Functional Enrichment of Differential Metabolites (DMs)

Metabolite importance in the PLS-DA loading was identified by variable importance in the projection (VIP) scores. The Student’s *t*-test was used to assess the statistical significance of DMs. Benjamini and Hochberg (BH) methods were implemented to control the false discovery rate (FDR). DMs with fold change (FC) ≥ 2 or ≤1/2 (absolute value of the log2ratio ≥ 1), VIP > 1.0, and FDR < 0.001 were considered to be significant.

To further understand the preferential function of DMs, KEGG enrichment of DMs was implemented in MBRole 2.0 software (http://csbg.cnb.csic.es/mbrole/, accessed on 27 December 2021). Rigorous FDR values  <  0.01 were considered to be significant.

### 2.9. Data Analysis

Data of mitochondrial swelling and enzymatic activity were analyzed using GraphPad Prism 8.0.2 (GraphPad Software Inc., La Jolla, CA, USA). The results were presented as mean ± standard deviation (S.D.) of three independent replicates. Student’s *t*-test was used to evaluate between-group variance. *p* values < 0.05 were considered to be significant.

## 3. Results

### 3.1. Acquirement and Verification of the Isolated Mitochondria

**Figure 1:**
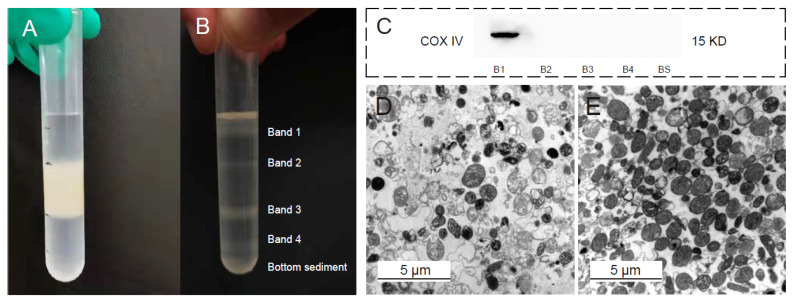
Acquisition and verification of the purity of the isolated mitochondria. (**A**) Typical appearance of the centrifuge tube following layering of the crude mitochondrial fraction. (**B**) Typical appearance of the centrifuge tube at the conclusion of the density gradient centrifugation step. (**C**) The purity of the mitochondria was confirmed by Western blot. TEM visualization of the crude (**D**) and purified (**E**) mitochondria.

The crudely extracted mitochondria were obtained from the fat body using differential centrifugation (Figure 1A). After discontinuous Nycodenz density gradient centrifugation, the cell constituents in the centrifuge tube were divided into four layers (bands), excluding bottom sediments (Figure 1B). Western-blot analysis showed that only the proteins in Band 1 were positive for the mitochondrial inner membrane marker COXIV (Figure 1C and Figure S1), whereas the proteins in Band 2–4 and bottom sediment were negative. This indicates that other subcellular organelles and cell impurities such as nuclear, sarcolemma, cytosol, and endoplasmic reticulum were not detected in the isolated mitochondria. Furthermore, TEM detection demonstrated the high purity and integrity of the isolated mitochondria (Figure 1E) compared with the crudely extracted mitochondria (Figure 1D).

### 3.2. Mitochondrial Swelling

**Figure 2:**
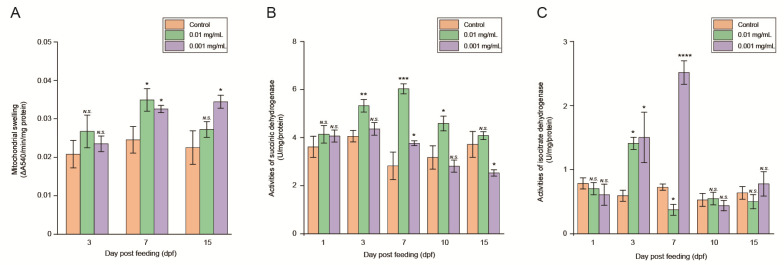
Evaluation of mitochondrial swelling and key enzyme activity in both 0.001 and 0.01 mg/mL abamectin-fed groups. (**A**) Detection of mitochondrial swelling in Blattella germanica fat body. (**B**) Detection of succinic dehydrogenase (SDH) activities of mitochondria. (**C**) Isocitrate dehydrogenase (IDH) activities of mitochondria. Results are shown as mean ± S.D. (n = 3). Compared with the control group: **** *p* < 0.00001, *** *p* < 0.0001, ** *p* < 0.001, * *p* < 0.05, *N.S.* denotes not significant.

The quantification results showed that mitochondrial swelling did not vary significantly in the 0.01 mg/mL abamectin-fed group at 3 dpf compared to the control (*p* > 0.05) (Figure 2A). Mitochondria swelling significantly increased by 42% at 7 dpf compared to the control (0.0349 ± 0.0029 at 7 dpf vs. 0.0245 ± 0.0035 in the control, *p* < 0.05). Mitochondrial swelling did not present significant changes compared to control (*p* > 0.05).

At 3 dpf, the mitochondrial swelling in the 0.001 mg/mL abamectin-fed group did not differ significantly compared to the control (*p* > 0.05, Figure 2A). The mitochondrial swelling significantly increased by 32% and 53% at 7 and 15 dpf, respectively, compared with the control (0.0326 ± 0.0009 at 7 dpf vs. 0.0245 ± 0.0035 in the control, *p* < 0.05; 0.0344 ± 0.0017 at 15 dpf vs. 0.0225 ± 0.0044 in the control, *p* < 0.05).

### 3.3. Activity of SDH and IDH

SDH activity was not significantly different between 1 dpf and the control (*p* > 0.05) the 0.01 mg/mL abamectin-fed group (Figure 2B). When compared with the control, SDH activity increased by 31%, 113.83%, and 44.79% at 3, 7, and 10 dpf, respectively (5.3255 ± 0.2613 vs. 4.0556 ± 0.2372 at 3 dpf, *p* < 0.01; 6.0316 ± 0.2060 vs. 2.8224 ± 0.5753 at 7 dpf, *p* < 0.001; 4.5861 ± 0.3056 vs. 3.1718 ± 0.4857 at 10 dpf, *p* < 0.05). After that, activity of SDH did not show a significant difference at 15 dpf compared to the control (*p* > 0.05). In addition, SDH activity in the 0.001 mg/mL abamectin-fed group was not significantly different at 1 and 3 dpf compared to the control (*p* > 0.05). At 7 dpf, SDH activity significantly increased by 34% compared with the control (3.7657 ± 0.1032 at 7 dpf vs. 2.8224 ± 0.5753 in the control, *p* < 0.05). SDH activity was not significantly different at 10 dpf compared to the control (*p* > 0.05). At 15 dpf, SDH activity significantly decreased by 32% compared with the control (2.5303 ± 0.1317 at 15 dpf vs. 3.7169 ± 0.5369 in the control, *p* < 0.05).

IDH activity in the 0.01 mg/mL abamectin-fed group (Figure 2C) did not differ significantly at 1 dpf from that of control (*p* > 0.05). When compared with the control, IDH activity increased by 137% at 3 dpf (1.4100 ± 0.9430 at 3 dpf vs. 0.5937 ± 0.0855 in the control, *p* < 0.05). At 7 dpf, IDH activity decreased by 48% compared with that of the control (0.3745 ± 0.0854 at 7 dpf vs. 0.7271 ± 0.0480 in the control, *p* < 0.05). Thereafter, IDH activity did not change significantly compared with that of the control (*p* > 0.05). Furthermore, IDH activity in the 0.001 mg/mL abamectin-fed group did not vary significantly at 1 dpf compared to the control (*p* > 0.05). When compared with the control, IDH activity increased by 152% and 246%, at 3 and 7 dpf, respectively (1.5012 ± 0.3933 vs. 0.5937 ± 0.0855 at 3 dpf, *p* < 0.05; 2.5164 ± 0.1834 vs. 0.7271 ± 0.0480 at 7 dpf, *p* < 0.0001). Subsequently, IDH activity did not differ significantly compared to that of the control (*p* > 0.05).

### 3.4. Mitochondrial Metabolics

#### 3.4.1. Overview of Sequencing Data

**Figure 3:**
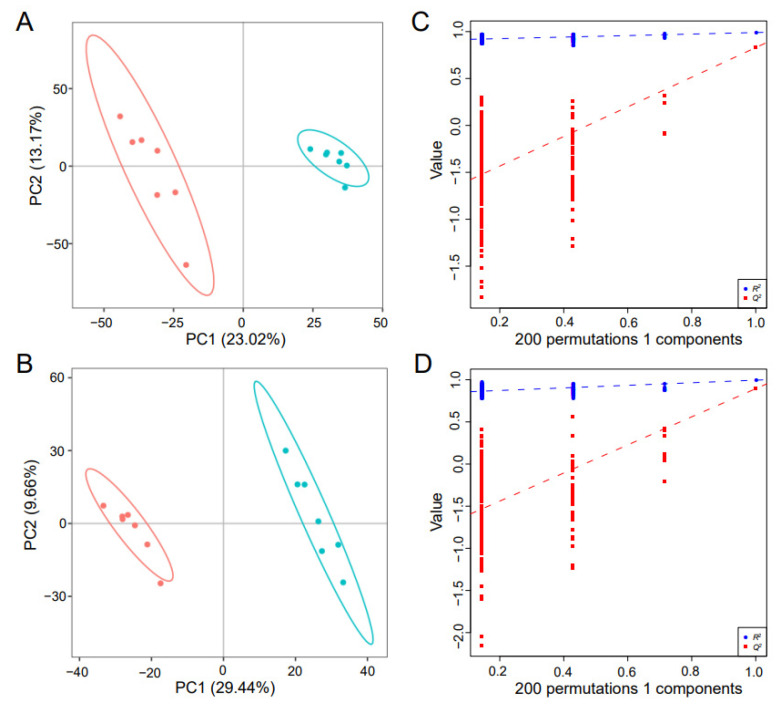
Score scatter plots and validation plots of PLS-DA of the abamectin-fed and the control groups. PLS-DA score scatter plot in PIM (**A**) and NIM (**B**). PLS-DA model validation plot in PIM (**C**) and NIM (**D**). Red solid circles indicate the control, while blue those indicate the abamectin-fed groups.

Total icon Chromatogram (TIC) spectra of 14 samples presented high consistency and abundant material peaks in PIM (Figure S2A) and NIM (Figure S2B) across retention time, indicating a reliable mass spectrographic analysis. In PIM and NIM, the number of metabolic ions is shown in Table S1. Since several metabolites included multiple isomers, only one representative for multiple metabolic ions belonging to the same metabolite was used for downstream analysis. Finally, in total, 3389 and 1673 metabolites were annotated in PIM and NIM, respectively. In the HMDB database, 315 and 195 metabolic ions were annotated in PIM and NIM, respectively. In the KEGG database, 316 and 218 metabolic ions were annotated in PIM and NIM, respectively. PCA analysis showed that seven replicates of the control groups clustered together in both PIM (Figure S3A) and NIM (Figure S3B) and were separated from the cluster of the 0.001 mg/mL abamectin-fed groups, indicating reliable experimental treatments. Similar clusters were further demonstrated by PLS-DA analysis in both PIM (Figure 3A) and NIM (Figure 3B). The R^2^ and Q^2^ values of intercepts demonstrated a reliable PLS-DA model without overfitting in both PIM (R^2^ = 0.9088, Q^2^ = −0.7491, Figure 3C) and NIM (R^2^ = 0.8421, Q^2^ = −0.7729, Figure 3D). The PLS-DA models displayed a strong goodness of fit and high predictability for metabolic analysis.

#### 3.4.2. DMs and Their KEGG Pathways

**Figure 4:**
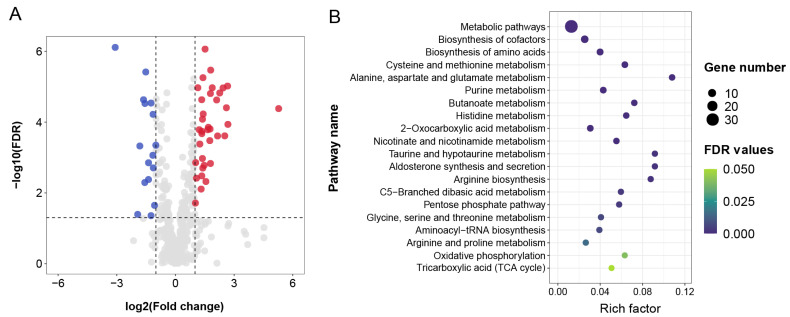
(**A**) A volcano plot of DMs. The red dots indicate significant up-regulation; the blue dots indicate significant down-regulation; the gray dots indicate no significance. (**B**) Twenty enriched KEGG pathways typically involved in toxic responses to abamectin feeding. Scatter plot of KEGG enrichment in DMs.

A total of 52 DMs were identified between the abamectin-fed and the control groups, including 36 up- and 16 down-regulated DMs (Figure 4A). These DMs are listed in Table 1 in detail. The top three most up-regulated DMs were 2-oxoglutarate, followed by D-glucosylsphingosine, and adenosine 2′-phosphate (ADP). Conversely, the top three most down-regulated DMs were 2-hydroxymalonate, followed by thr-arg and 5-hydroxyisourate. Several DMs were related to amino acid metabolism (e.g., d-proline, thr-arg, and leucine), nucleotide metabolism (e.g., adenosine, adenosine 5′-monophosphate (AMP), and nicotinamide adenine dinucleotide (NADH)), lipid metabolism (e.g., decanoylcarnitine, 9,12-hexadecadienoylcarnitine, and l-octanoylcarnitine), and carbohydrate metabolism (e.g., α-KG, 2-dehydro-d-gluconate, and 3-phospho-d-glyceric acid (3-PGA)).

Furthermore, DMs were significantly enriched in 39 KEGG pathways (Table S2) such as metabolic pathways (ko01100), biosynthesis of secondary metabolites (ko01110), and biosynthesis of cofactors (ko01240). According to previous studies [24,30,42,43], 20 pathways (Figure 4B) have been reported to be typically associated with toxic responses. Among them, eight KEGG pathways were involved in amino acid metabolism, such as cysteine and methionine metabolism (ko00460), alanine, aspartate and glutamate metabolism (ko00250), histidine metabolism (ko00340); four KEGG pathways were associated with carbohydrate metabolism, including butanoate metabolism (ko00650), C5-branched dibasic acid metabolism (ko00660), TCA cycle (ko00020), and pentose phosphate pathway (PPP) (ko00030); and eight pathways were involved in global and overview maps (e.g., metabolic pathways (ko01100)), metabolism of cofactors (e.g., biosynthesis of cofactors (ko01240)), nucleotide metabolism (e.g., purine metabolism (ko00230)), and energy metabolism (e.g., oxidative phosphorylation (ko00190)).

#### 3.4.3. Working Model of Key Energy Metabolism-Related Metabolites (KEMMs) in the Toxic Response of Mitochondria to Abamectin Feeding

**Figure 5:**
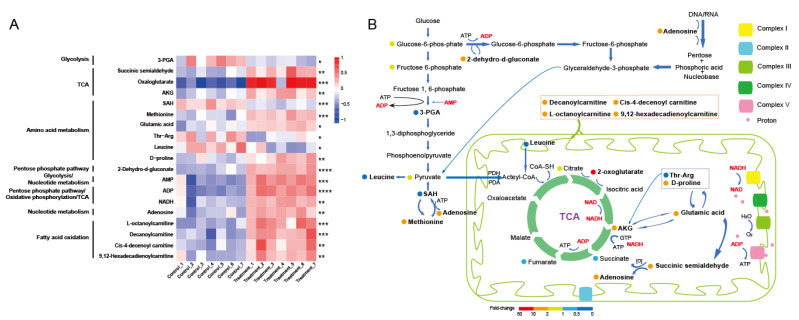
Working model of key energy metabolism-related metabolites (KEMMs) in the toxic response of mitochondria to abamectin feeding. (**A**) Heatmap of KEMMs. Compared with control groups, up-regulation was marked by red, while down-regulation was marked by blue in treatment groups. **** *FDR* < 0.00001, *** *FDR* < 0.0001, ** *FDR* < 0.001, * *FDR* < 0.05. (**B**) Working model of KEMMs. Metabolites identified in this study were marked by filled color circles. The red and orange circles represent up-regulation in treatment groups compared with control groups, while dark blue circles present down-regulation. Light yellow and sky-blue colored circles represent no significance in fold change of metabolites. No marked circles represent the metabolites that were not were identified in this study, but reported in other animals. This figure was adapted from Figure 2B in [30].

Referring to earlier studies [24,30,42,43], 19 key energy metabolism-related metabolites (KEMMs) were further detected in the list of DMs depicted in Table 1. Their heatmap is illustrated in Figure 5A. Compared with the control, 15 KEMMs were identified to be up-regulated in 0.01 mg/mL abamectin-fed group, including 2-oxoglutarate (up-regulated by 38.74-fold), ADP (6.34-fold), AMP (6.09-fold), decanoylcarnitine (4.80-fold), cis-4-decenoyl carnitine (4.45-fold), L-octanoylcarnitine (4.31-fold), 2-dehydro-d-gluconate (3.49-fold), succinic semialdehyde (3.42-fold), NADH (3.38-fold), 9,12-hexadecadienoylcarnitine (3.21-fold), adenosine (3.15-fold), L-methionine (2.61-fold), α-KG (2.55-fold), d-proline (2.37-fold), and glutamic acid (2.05-fold). On the other hand, four KEMMs were identified to be down-regulated, including 3-PGA (0.33-fold), leucine (0.38-fold) thr-arg (0.48-fold), and SAH (0.45-fold). A working model of all these 19 KEMMs in responses to abamectin feeding was constructed and is shown in Figure 5B. The results showed that the KEMMs primarily participated in ATP production and energy metabolism-related pathways such as the TCA cycle, glycolysis, OXPHOS, PPP, amino acid metabolism, and β-oxidation.

## 4. Discussion

Given the importance of mitochondria isolation to understand their roles in toxic responses at the subcellular level, we isolated high-purity and -integrity mitochondria from *B. germanica* fat body fed with abamectin. The current study showed no substantial (although modest) increase in mitochondrial swelling at 3 dpf under both abamectin-fed concentrations. At 7 and 15 dpf, abamectin obviously induced the most serious damage to mitochondria (i.e., swelling). Previous studies have similarly reported a massive swelling alteration of mitochondria in the brain of *Nilaparvata lugens* challenged with fipronil [44] and the gills of *Ruditapes philippinarum* exposed to ammonia nitrogen [18]. In general, the occurrence of abnormal mitochondrial phenotype can be linked to functional changes in energy metabolism [45,46]. These changes may also impact their normal bioenergetic activity (e.g., abnormal ATP production) [18,46]. This evidence indicates that abamectin feeding at the two concentrations used in this study effectively induced a toxic response of the mitochondria of *B. germanica* fat body, which was likely accompanied by alteration in energy metabolism.

SDH is a marker enzyme of the mitochondrial inner membrane whose activation reflects changes in energy metabolism [47,48]. In this study, the activation of SDH significantly increased at 7 dpf upon abamectin stress, suggesting an alteration in mitochondrial energy metabolism. In the case of *S. yangtsekiense*, the increased activity of SDH with elevated Cd concentration can be interpreted as an enhanced ability to generate energy to cope with Cd-induced mitochondrial injury [42]. Therefore, an enhancement in SDH activity suggests that apoptosis occurred, removing injured mitochondria and requiring energy consumption upon abamectin stress. However, as compared with the control, SDH activity decreased at 15 dpf under 0.001 mg/mL abamectin. Inhibition of SDH activity is a key indicator for the attenuation of mitochondrial respiration and energy production [47,49], which disturbed electron transport and caused cellular energy deficits [49]. In addition, as an ADP-activated allosteric enzyme in mitochondria, IDH is also important for maintaining normal aerobic metabolism via the TCA cycle, which catalyzes isocitrate decarboxylation to α-KG, with the reduction of NADP to NADPH [50]. IDH activity extremely increased at 7 dpf in 0.001 mg/mL abamectin-fed group. Previous studies demonstrated an enhancement in the TCA cycle induced by IDH in early-staged *Danio rerio* individuals exposed to nanopesticides [30]. Therefore, the increase of IDH activity promoted aerobic metabolism through the TCA cycle upon abamectin stress, with increasing energy demand in *B. germanica* fat body. IDH activity significantly changed at 7 dpf in the abamectin-fed group compared with that of the control group, indicating that mitochondria of *B. germanica* fat body are likely to impact on the ability of energy metabolism. Compared with the control, IDH activity exhibited a slight (non-significant) variation at 10 and 15 dpf, suggesting a possible disturbance alleviation in mitochondrial energy metabolism over time.

Furthermore, we generated the quality metabolite profiles of the isolated mitochondria from the fat body of *B. germanica* fed abamectin. A set of DMs involved in the toxic responses was identified. In particular, 2-oxoglutarate, a metabolic intermediate in the TCA cycle, was shown to have the most increasing level in all DMs. This metabolite served as a biomarker to assess hepatotoxicity and injury of the mitochondrial respiratory function, which is closely linked to energy metabolism and induced by aflatoxin B1 [51]. Moreover, many amino acids served as precursors of intermediates in energy metabolism [30,52]. D-proline and lutamine can be converted into the amino acid glutamate through glutaminase, which can be then converted into α-ketoglutarate through transaminase and glutamate dehydrogenase and ultimately enter the TCA cycle to produce energy [30,52]. It has been reported that amino acids participate as fuels in the toxic response of *D. rerio* to nanopesticides [30]. The metabolic levels of glutamic acid and D-proline in this study demonstrate the effect of abamectin feeding on amino acid-based energy metabolism in the mitochondria of *B. germanica* fat body. Fatty acids in the form of acyl-carnitine esters entered the mitochondria for oxidation, resulting in higher efficiency of the TCA cycle and energy production [53,54]. In the present, metabolic levels of four acyl-carnitine species, which are key amino acid analogs involved in fatty acid-based energy metabolism, increased compared with the control. Therefore, the increasing levels of carnitines may be a sign of changes in mitochondrial energy metabolism triggered by abamectin feeding. In addition, nucleotides and their analogs (e.g., adenosine, ADP, and AMP) were also found in the list of DMs in this study. The nucleotide metabolism alteration compensated for the decrease of energy metabolism in the toxicity of difenoconazole to *D. rerio* individuals of early stage, allowing organismic metabolism homeostasis [55].

At the metabolic pathway levels, eight KEGG pathways enriched by DMs were associated with amino acid metabolism. In addition, aminoacyl-tRNA biosynthesis pathway plays a central role in matching amino acids with tRNAs that contain the corresponding anticodon [56]. Therefore, abamectin affected amino acid and protein levels in the mitochondria of *B. germanica* fat body. Metabolic pathways of amino acids and proteins are involved in detoxification and energy compensation in *Sepia pharaonis* under ammonia nitrogen [57]. Triphenyl phosphate exposure influenced carbohydrate metabolic pathways in *D. rerio* liver [58]. As the most key carbohydrate metabolic pathway [59], the TCA cycle was enriched by DMs in the present study. Depletion of the TCA cycle intermediate pool and impaired energy-producing metabolism promoted high glucose/palmitate (HG/PA)-induced cytotoxicity to beta cells [59]. In addition, nucleotide metabolic pathways were shown to be directly linked to cellular homeostasis required for energy production and metabolism, which was basically for normal carbohydrate metabolism, oxidative phosphorylation, and signal transduction [60,61]. The current study found that abamectin feeding influenced nucleotide metabolism-related pathways in the mitochondria of abamectin-fed *B. germanica* fat body. These findings suggest that abamectin feeding affected amino acid, carbohydrate, and nucleotide metabolic pathways-mediated energy metabolism of mitochondria in *B. germanica* fat body, further demonstrating the energy metabolism-related toxic response of mitochondria from the perspective of metabolic pathways.

As shown in the working model constructed for KEMMs, energy metabolism starts with glycolysis/glucose that can be converted into pyruvate. Abamectin reduced 3-PGA (intermediate metabolites of glycolysis), but increased the level of 2-dehydro-d-gluconate (intermediate metabolites of PPP). Thus, abamectin feeding probably inhibited glycolysis, while improving PPP. The combination of PPP and glycolysis generated essential compounds for ATP production and consumption [62]. Isocitric acid in combination with NAD(+) formed NADH that can be converted into α-KG and CO2 under the action of IDH [50]. α-KG plays a decisive role in the overall rate of the TCA cycle [50]. Abamectin feeding resulted in increased α-KG and NADH, as well as increased enzyme activity of IDH, suggesting that abamectin altered energy production by promoting the TCA cycle in the mitochondria of *B. germanica* fat body. This also can be further determined by an increasing level of intermediate metabolites of TCA cycle (2-oxoglutarate and succinic semialdehyde). NADH is an essential participant in energy metabolism at the three stages of cellular respiration, i.e., glycolysis, TCA cycle, and OXPHOS [61,63]. An increasing level of NADH may be attributed to metabolic changes in the TCA cycle and OXPHOS. Thereafter, the OXPHOS contains several key intermediates/basic components used for energy metabolism, such as adenosine, ADP, and AMP [30]. Recent studies also found that R-(−)-dinotefuran induced adenosine and ADP [64]. Therefore, with the increase of ADP and AMP in the OXPHOS process, abamectin feeding increased ATP consumption in mitochondria. In addition, the working model showed that abamectin induced L-methionine (intermediate metabolites of methionine metabolism), a precursor of S-adenosyl-L-methionine (SAM). An increasing L-methionine promoted SAM accumulation [65]. Subsequently, SAH, a potent inhibitor of SAM-dependent methylation reaction, can be formed by demethylation of SAM [65]. Abamectin feeding decreased SAH while increasing ATP consumption, indicating that SAM-dependent methylation and energy metabolism were enhanced. Furthermore, increasing proline, which is a common osmotic effector, was beneficial for cell osmotic pressure and antioxidation upon adverse stress [57]. As one of the most versatile amino acids, arginine was metabolically interconvertible with proline and glutamic acid [52]. Abamectin feeding induced D-proline and glutamic acid levels, while decreasing thr-arg levels, indicating changes in amino acid metabolism, osmotic pressure, and antioxidation capacity. Changes in these cellular functions have certainly an impact on mitochondrial energy metabolism. Acetyl-CoA is the primary metabolic end product of leucine [66]. Thus, abamectin-induced leucine reduction may result in a drop in acetyl-CoA levels, impacting the TCA cycle. Mitochondria are recognized as the primary organelles where β-oxidation of fatty acid takes place [53,54,67]. The current study uncovered the working model of β-oxidation of fatty acids containing one long-chain acyl-carnitine (9,12-hexadecadienoylcarnitine) and three medium-chain acyl-carnitines (decanoylcarnitine, cis-4-decenoyl carnitine, and L-octanoylcarnitine).

## 5. Conclusions

In summary, the current study is the first to focus on the toxic response of the isolated mitochondria from multiple perspectives (i.e., morphology, physiology, metabolites, and metabolic pathways). High-purity mitochondria were successfully isolated from *B. germanica* fat body. Abamectin feeding induced swelling in the mitochondrial membrane, as well as changes in the activities of SDH and IDH as mitochondrial energy metabolism-related enzymes, confirming our hypothesis. Furthermore, changes in metabolites and metabolic pathways typically involved in mitochondrial energy metabolism were also analyzed. It is worth noting that several metabolites found to be involved in abamectin stress cannot be reasonably linked to toxic responses. Nonetheless, these metabolites indeed have toxic responses to abamectin stress in the mitochondria of *B. germanica* fat body. Further investigations using experimental biology are required to confirm the metabolites identified in this study.

## Figures and Tables

**Table 1:** List of differential metabolites (DMs) in the fat body mitochondria between control and *B. germanica*-fed abamectin. # Key energy metabolism-related metabolites (KEMMs).

ID	Metabolites	Ratio	FDR	VIP
**Up-regulated differential metabolites**				
#neg-1.553_204.02686	2-oxoglutarate	38.74	4.15 × 10^−5^	1.60
neg-4.753_461.33574	d-glucosylsphingosine	6.42	1.16 × 10^−4^	1.65
#pos-0.967_347.06211	adenosine 2′-phosphate	6.34	9.57 × 10^−6^	2.12
#pos-1.456_347.06211	adenosine 5′-monophosphate	6.09	3.92 × 10^−5^	2.12
neg-0.919_454.10891	n-{3-[(3,5-dimethoxyphenyl)amino]-2-quinoxalinyl}-4-fluorobenzenesulfonamide	5.74	2.44 × 10^−4^	1.61
pos-0.9_165.04525	l-methionine sulfoxide	5.41	1.08 × 10^−5^	2.10
#pos-4.252_315.23979	decanoylcarnitine	4.80	1.50 × 10^−5^	2.08
#pos-4.161_313.22394	cis-4-decenoyl carnitine	4.45	2.46 × 10^−4^	1.97
#pos-4.027_287.20846	l-octanoylcarnitine	4.31	2.37 × 10^−5^	2.06
neg-1.099_566.05549	uridine 5′-diphosphogalactose	3.69	1.07 × 10^−5^	1.77
#neg-0.885_194.04251	2-dehydro-d-gluconate	3.49	3.40 × 10^−6^	1.84
pos-4.265_429.3077	hexadecanedioic acid mono-l-carnitine ester	3.46	1.47 × 10^−3^	1.74
neg-4.781_228.20891	myristic acid	3.46	1.56 × 10^−5^	1.74
#neg-1.445_102.03159	succinic semialdehyde	3.42	1.57 × 10^−4^	1.63
#pos-1.46_663.10772	nicotinamide adenine dinucleotide	3.38	3.34 × 10^−4^	2.02
#pos-4.705_395.30215	9,12-hexadecadienoylcarnitine	3.21	1.40 × 10^−4^	1.98
#pos-0.971_267.09595	adenosine	3.15	1.64 × 10^−4^	2.04
pos-7.609_306.25481	8z,11z,14z-eicosatrienoic acid	2.94	4.76 × 10^−3^	1.66
neg-0.919_440.09346	1-deoxy-1-{2,6,8-trioxo-7-[3-(phosphonooxy)propyl]-1,2,3,6,7,8-hexahydro-9h-purin-9-yl}-d-ribitol	2.86	8.66 × 10^−7^	1.88
pos-0.743_111.07941	histamine	2.77	1.66 × 10^−3^	1.72
pos-3.562_158.07292	2-methoxynaphthalene	2.68	5.90 × 10^−5^	2.07
neg-0.919_437.09769	4-amino-1-(6-deoxy-6-phosphono-beta-d-allofuranosyl)-5-(phenylethynyl)-2(1h)-pyrimidinone	2.65	5.56 × 10^−6^	1.83
neg-1.995_465.10551	1-benzyl-3-[(e)-{[2,3,5,6-tetrafluoro-4-(trifluoromethyl)phenyl]hydrazono}methyl]-1h-indole	2.63	1.99 × 10^−3^	1.38
#neg-1.111_149.05098	l-methionine	2.61	8.27 × 10^−5^	1.68
pos-3.545_157.07358	3-methylcrotonylglycine	2.60	1.06 × 10^−3^	1.78
#neg-0.99_146.02142	α-ketoglutaric acid	2.55	2.11 × 10^−4^	1.61
neg-0.99_100.01596	succinic anhydride	2.55	1.82 × 10^−4^	1.62
neg-3.689_924.30452	disialyllactose	2.55	3.31 × 10^−3^	1.40
neg-0.954_174.10019	n5-ethyl-l-glutamine	2.54	2.33 × 10^−5^	1.73
pos-1.057_249.03013	norepinephrine sulfate	2.49	7.77 × 10^−3^	1.70
#neg-0.948_115.06322	d-proline	2.37	4.16 × 10^−4^	1.56
pos-1.448_132.02413	3-methylsulfolene	2.32	1.63 × 10^−4^	1.99
neg-0.922_342.11649	1-alpha-d-galactosyl-myo-inositol	2.21	1.07 × 10^−5^	1.76
neg-3.452_234.01978	4-ethyl-2,6-dihydroxyphenyl hydrogen sulfate	2.11	3.84 × 10^−3^	1.44
#neg-0.879_87.03192	glutamic acid	2.05	1.39 × 10^−3^	1.61
pos-3.563_150.07897	6-methylnicotinamide	2.03	1.93 × 10^−2^	1.45
**Down-regulated differential metabolites**				
pos-4.587_162.06745	4-hydroxycinnamoylmethane	0.12	7.74 × 10^−7^	2.18
pos-0.913_345.11615	isofenphos	0.26	4.04 × 10^−2^	1.41
pos-0.967_179.06119	cyclamic acid	0.28	4.72 × 10^−4^	1.98
pos-0.966_384.12064	oxyisocyclointegrin	0.32	2.33 × 10^−5^	2.08
#pos-0.917_185.99236	3-phospho-d-glyceric acid	0.34	5.09 × 10^−3^	1.72
pos-0.966_192.06033	(2e)-2-(methylthiomethyl)-3-phenyl-2-propenal	0.34	2.97 × 10^−5^	2.08
pos-5.869_453.28422	glycerophospho-n-palmitoyl ethanolamine	0.35	3.84 × 10^−6^	2.16
#pos-0.939_131.09429	leucine	0.38	4.19 × 10^−3^	1.67
neg-0.966_336.05708	thiamine	0.39	1.41 × 10^−3^	1.60
pos-7.197_467.33622	1-(beta-d-arabinofuranosyl)-4-(hexadecylamino)-2(1h)-pyrimidinone	0.42	2.89 × 10^−5^	2.07
neg-3.249_84.05752	3-methylbut-2-enal	0.42	4.38 × 10^−2^	1.07
neg-0.94_540.14607	(2r,3s,5r)-5-(4-amino-2-oxo-1(2h)-pyrimidinyl)-2-(hydroxymethyl)tetrahydro-3-furanyl [(2r,3s,5r)-5-(6-amino-9h-purin-9-yl)-3-hydroxytetrahydro-2-furanyl]methyl hydrogen phosphate	0.45	8.64 × 10^−4^	1.64
#pos-1.569_384.1207	s-adenosyl-l-homocysteine	0.45	6.02 × 10^−5^	2.05
neg-1.497_184.02311	5-hydroxyisourate	0.46	1.96 × 10^−3^	1.45
#pos-3.263_275.16036	thr-arg	0.48	2.24 × 10^−2^	1.39
neg-0.937_120.00574	2-hydroxymalonate	0.50	4.48 × 10^−4^	1.56

## Data Availability

All metabolomics data generated in this study has been submitted to MetaboLights repository (accession numbers: MTBLS4903).

## Supplementary Materials

The following supporting information can be downloaded at: https://www.mdpi.com/article/10.3390/insects13121091/s1, Supplementary methods: Mitochondria isolation, Western blot analysis; Figure S1. The original image of Western analysis corresponding to Figure 1C; Figure S2. Total icon Chromatogram (TIC) spectra of all the samples in PIM (A) and NIM (B) across retention time; Figure S3. PCA analysis for metabolic profiles of all the samples in PIM (A) and NIM (B); Table S1. Summary of metabolic ions and metabolites for each sample; Table S2. List of KEGG pathways enriched by differential metabolites, sorted by significance.
