# Supplementary material for: Subcellular-Level Mitochondrial Energy Metabolism Response in the Fat Body of the German Cockroach Fed Abamectin
Source: Insects. 2022 Nov 27;13(12):1091. doi: 10.3390/insects13121091 (PMC9782180; doi:10.3390/insects13121091)
Supplement: Supplementary file 1 [file insects-13-01091-s001.zip › insects-1962046-supplementary.pdf]

Supplementary Material

Supplementary methods

Mitochondria isolation: For each abamectin-challenged and control group, liver tissues (approximate 600 mg) of multiple individuals were mixed, transferred into a 7-mL Dounce homogenizer (Cat. No. 357542, Wheaton, New Jersey, USA) containing 4 mL isolation buffer (STE: 120 mM Tris, 15 mM EDTA-2K, 960 mM sucrose, pH 7.1, tissue wet wt/STE vol: 100 mg/mL). The pieces of tissue were homogenized on ice (ten up-and-down strokes with the loose glass pestle, and then fifteen strokes with the tight glass pestle) in Dounce homogenizer. The whole homogenization process was completed within 0.5 h. The homogenized tissue was then transferred to a 50 mL plastic centrifuge tube, and centrifuged at 1,000 × g at 4 ℃ for 5 min in a high-speed refrigerated centrifuge (Shuke, Sichuan, China). The supernatant was then transferred to another centrifuge tube. The residual precipitate was transferred into the Dounce homogenizer again after suspension using double volume STE, ground up with the tight glass pestle, and centrifuged in a 50 mL tube at 1,000 × g at 4 ℃ for 5 min. The supernatants obtained from the two runs of the centrifuge were pooled, centrifuged again in a 50-mL tube at 15,000 × g at 4 ℃ for 10 min and the supernatant discarded. The retained precipitate was assumed to be the considered as crudely-extracted mitochondria.

The crudely-extracted mitochondria were further separated from other cell constituents using a discontinuous Nycodenz gradient (Nycodenz reagent: Cat. No. 10206333, Alere Technologies AS, Oslo, Norway). Namely, the crude samples were resuspended using a 2.4 mL pre-cooled (4 ℃) Nycodenz solution (Nycodenz powder (mg): STE solution (mL) = 25%) in a 5-mL plastic centrifuge tube. The Nycodenz solutions at 34%, 30%, 25% (this density contains the crudely-extracted mitochondria), 23%, and 20% were used for the subsequent density gradient centrifuge treatments prepared in a 13 PA centrifuge tube (10 mL volume, Cat. No. 901062, Hitachi, Tokyo, Japan), in 1 mL, 1.6 mL, 2.4 mL, 1.6 mL, and 0.6 mL, respectively. Notably, each concentration of the Nycodenz solution was sequentially

added into the 13 PA tube, gently, to avoid any mixing. Finally, the Nycodenz solution at five density gradients was obtained, centrifuged at 120,000 × g at 4 ℃ for 1.5 h. Each separated band containing cell constituents was transferred to a 5-mL centrifuge tubes using a pipette with 4 times volume of STE, and centrifuged at 15,000 × g at 4 ℃ for 10 min.

Western blot analysis: The lysis of eight constituents (i.e., homogenate of insect tissues, the crudely-extracted mitochondria, isolated mitochondria, and each separated band in five density gradients) was carried out in a NP-40 buffer plus protease inhibitor (Cat. No.9016-45-9, VWR International, Pennsylvania, USA). During this period, the bottom of the tube was gently flicked every 5 min to improve protein dissolution. The Pierce® BCA Protein Assay Kit (Cat. No.23227, Thermo Scientific) was used to determine the protein concentration of cell constituents according to the manufacturer's instructions. The absorbance was measured at 562 nm using a microplate reader (Thermo Scientific). BSA-free samples were used as a blank control. The plotted standard curve was: $y = 1.026x + 0.0914$, $R^2 = 0.9907$.

Equal amounts of proteins (20 μg) were separated by 12% SDS-PAGE. After that, the separated proteins were transferred to PVDF membranes (Cat. No. IPVH00010, Biosharp, Hefei, China) using a wet transfer apparatus (Bio-Rad; California, USA) at 200 mA at 4 ℃ for 1.5 h. The proteins on the membranes were blocked with 5% skimmed milk powder in TBST Tris buffer (consisting of 1 M Tris-HCl Solution, 6.64 M NaCl, and 0.05% Tween-20) at room temperature for 4 h. The TBST Tris buffer was used to wash the membrane three times. The blots were then incubated with the rabbit ploymonoclonal anti-COX IV (cytochrome C oxidase subunit IV) antibody (1:800, Thermo Scientific, California, USA) at 4 ℃ overnight. After the membranes were washed with TBST three times, the membranes were incubated with horseradish peroxidase (HRP)-conjugated Goat anti-Rabbit IgG (1:100,000; Thermo Scientific), a secondary antibody, at room temperature for 1 h. After the membranes were washed with TBST three times, they were visualized with ECL Plus Western Blotting Detection Reagents (Cat. No. 32106, Thermo Scientific) using a Gel Documentation

System (Bio-Rad; California, USA). Gray values of the target bands were calculated using ImageJ, version 1.8.0 (NIH, Maryland, USA) to quantify their abundance.

Table S1. Summary of metabolic ions and metabolites for each sample.

Mode	Category	Control							Treatment						
		Ck_1	Ck_2	Ck_3	Ck_4	Ck_5	Ck_6	Ck_7	T_1	T_2	T_3	T_4	T_5	T_6	T_7
PIM	Metabolic ions	4253	4253	4253	4253	4253	4253	4253	4253	4253	4253	4253	4253	4253	4253
	All metabolites							3389							
	HMDB annotated metabolic ions							464							
	KEGG annotated metabolic ions							449							
	HMDB annotated metabolites							315							
	KEGG annotated metabolites							316							
NIM	Metabolic ions	1881	1881	1881	1881	1881	1881	1881	1881	1881	1881	1881	1881	1881	1881
	All metabolites							1673							
	HMDB annotated metabolic ions							246							
	KEGG annotated metabolic ions							273							
	HMDB annotated metabolites							195							
	KEGG annotated metabolites							218							

Table S2. List of KEGG pathways enriched by differential metabolites, sorted by significance.

Pathways	Id	FDR
Metabolic pathways	ko01100	8.41E-16
Biosynthesis of secondary metabolites	ko01110	0.000013
Biosynthesis of cofactors	ko01240	2.28E-05
Biosynthesis of alkaloids derived from histidine and purine	ko01065	3.13E-05
Cyanoamino acid metabolism	ko00460	5.77E-05
Biosynthesis of amino acids	ko01230	0.000164
Cysteine and methionine metabolism	ko00270	0.000191
Biosynthesis of various secondary metabolites	ko00997	0.000221
Alanine, aspartate and glutamate metabolism	ko00250	0.00041
FoxO signaling pathway	ko04068	0.000528
Purine metabolism	ko00230	0.000605
Butanoate metabolism	ko00650	0.000936
Carbon metabolism	ko01200	0.000948
Morphine addiction	ko05032	0.000948
Histidine metabolism	ko00340	0.001023
GABAergic synapse	ko04727	0.001023
Protein digestion and absorption	ko04974	0.001023
cGMP-PKG signaling pathway	ko04022	0.00114
2-Oxocarboxylic acid metabolism	ko01210	0.001184
ABC transporters	ko02010	0.001216
Nicotinate and nicotinamide metabolism	ko00760	0.001216
Synaptic vesicle cycle	ko04721	0.001414
D-Glutamine and D-glutamate metabolism	ko00471	0.001573
Glyoxylate and dicarboxylate metabolism	ko00630	0.001573
Regulation of lipolysis in adipocytes	ko04923	0.001782
Biosynthesis of alkaloids derived from ornithine, lysine and nicotinic acid	ko01064	0.001826
Taurine and hypotaurine metabolism	ko00430	0.003744
AMPK signaling pathway	ko04152	0.003744
Aldosterone synthesis and secretion	ko04925	0.003744
Arginine biosynthesis	ko00220	0.003912
cAMP signaling pathway	ko04024	0.004525
Glucagon signaling pathway	ko04922	0.004791
C5-Branched dibasic acid metabolism	ko00660	0.007841
Pentose phosphate pathway	ko00030	0.007979
Glycine, serine and threonine metabolism	ko00260	0.007979
Aminoacyl-tRNA biosynthesis	ko00970	0.007999
Arginine and proline metabolism	ko00330	0.008016
Oxidative phosphorylation	ko00190	0.009099
Citrate cycle (TCA cycle)	ko00020	0.009099

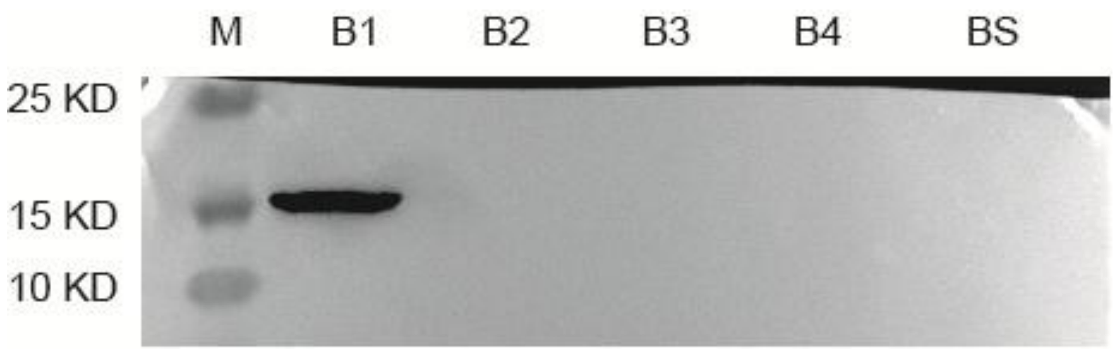

Figure S1. The original image of Western analysis corresponding to Figure 1C.

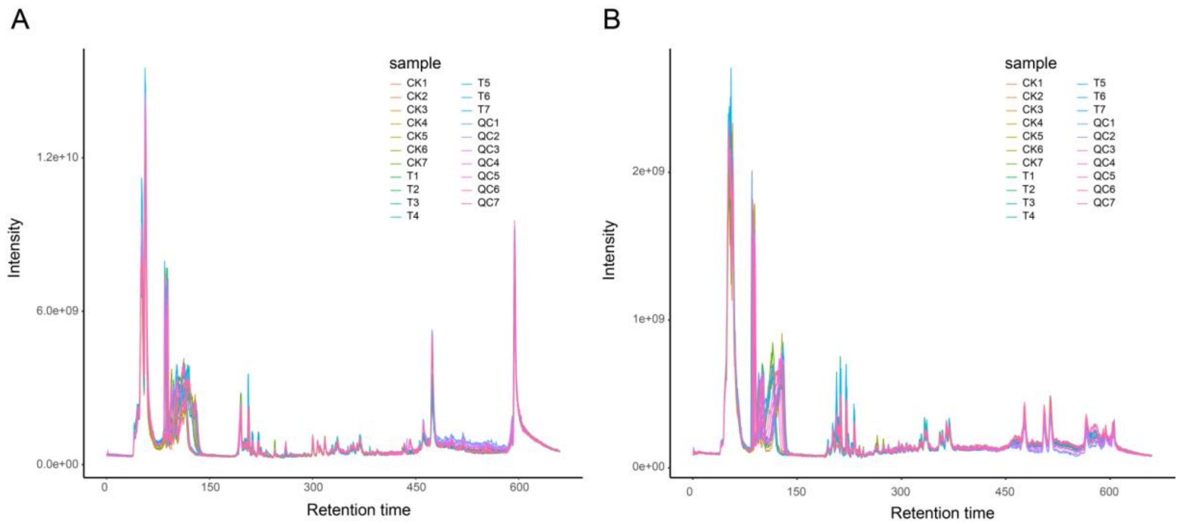

Figure S2. Total icon Chromatogram (TIC) spectra of all the samples in PIM (A) and NIM (B) across retention time.

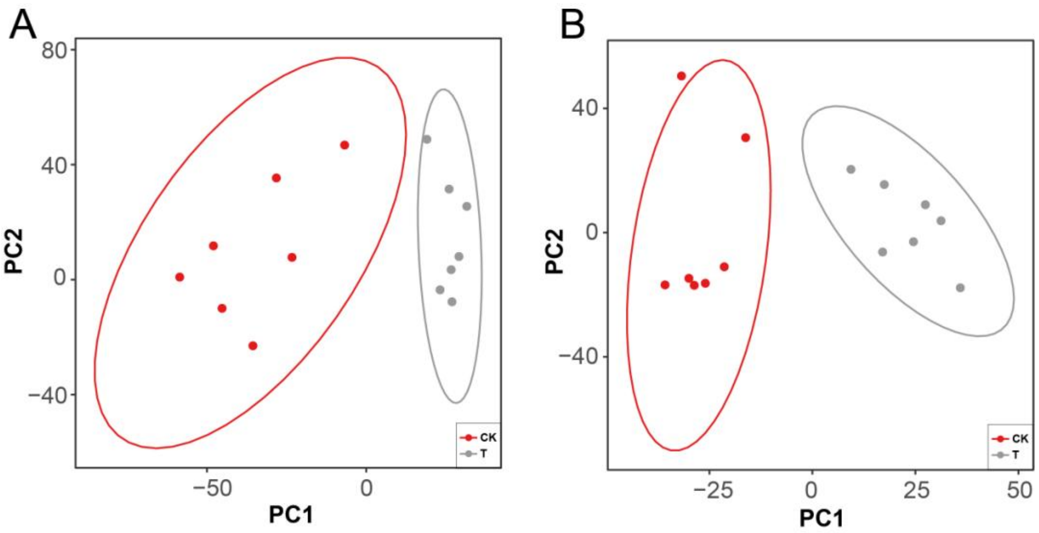

Figure S3. PCA analysis for metabolic profiles of all the samples in PIM (A) and NIM (B).
